# Mainstream Germline Genetic Testing with Expanded Eligibility for Early Breast Cancer Patients in a Large Integrated Health System

**DOI:** 10.1245/s10434-024-16223-7

**Published:** 2024-09-18

**Authors:** Veronica Shim, Audrey Karlea, Leslie Manace Brenman, Jamila Gul, Elizabeth Hoodfar, Tracy D. Chan, Poline C. Engeman, Vanessa M. Sheldon, Deirdre M. Thorne-Hadfield, Patience Odele, Brooke Vuong, Jennifer McEvoy, C. K. Chang, Dinesh Kotak, Laurel A. Habel

**Affiliations:** 1 Department of Surgery, Kaiser Permanente Oakland Medical Center, Oakland, CA USA; 2 Department of Genetics, Oakland, Kaiser Permanente, Oakland, USA; 3 Division of Research, Oakland, Kaiser Permanente, Oakland, USA; 4 Department of Genetics, Sacramento, Kaiser Permanente, Sacramento, USA; 5 Department of Surgery, South Sacramento, Kaiser Permanente, Sacramento, USA; 6 Department of Surgery, Central Valley, Kaiser Permanente, Modesto, USA; 7 Department of Radiology, San Rafael, Kaiser Permanente, San Rafael, USA; 8 Department of Surgery, San Rafael, Kaiser Permanente, San Rafael, USA; 9 Department of Medical Oncology, San Rafael, Kaiser Permanente, San Rafael, USA

**Keywords:** Mainstream germline genetic testing, Breast cancer, Pathogenic/likely pathogenic variant, Breast cancer-related gene, Multigene panel

## Abstract

**Background:**

This study evaluated a new mainstream genetic testing pathway for hereditary cancer, with expanded eligibility for early-stage breast cancer patients.

**Methods:**

The study compared multigene panel (62 genes) germline testing uptake and results for breast cancer patients at 4 pilot sites (*n* = 502 patients) and 10 non-pilot sites (*n* = 1792 patients) within Kaiser Permanente Northern California from December 2020, to June 2021. At the pilot sites, breast care coordinators (BCCs) offered and consented patients for testing, with eligibility expanded to include all patients age 65 years or younger. At the non-pilot sites, eligible patients were referred to genetics for pre-test counseling, ordering, and follow-up evaluation with the standard guideline that included all patients age 45 years or younger.

**Results:**

Demographic and disease characteristics were similar at the pilot and non-pilot sites. At the pilot verses non-pilot sites, a higher percentage of patients was tested overall (61.6% vs 31.7%) and across all age groups. The median time from breast biopsy to test result also was reduced (22 vs 33 days, respectively). A higher percentage of patients at the pilot sites was identified as having a pathogenic/likely pathogenic variant (PV/LPV) in a breast cancer-related gene (3.6% vs 1.6%). Although the percentage of total patients tested was nearly twofold higher at the pilot sites than at the non-pilot sites, the percentage of total patients seen by genetics was estimated to be similar (33.7% vs 31.7%).

**Conclusion:**

Mainstream genetic testing of breast cancer patients facilitated by BCCs makes it feasible for a large health care system to expand germline genetic testing to early breast cancer patients age 65 years or younger.

Multiple different guidelines are available for multigene panel germline testing for hereditary cancer in early breast cancer patients.^1–5^ The 2019 National Comprehensive Cancer Network (NCCN) guidelines recommended testing of all breast cancers diagnosed in patients age 45 years or younger, all triple-negative breast cancers diagnosed in patients age 60 years or younger, all breast cancer patients with Ashkenazi Jewish ancestry or a strong family history, and all breast cancer patients who are males at birth. Since 2019, the Society of American Breast Surgeons has advocated testing of all breast cancer patients based on a study showing that nearly half of patients who have breast cancer with a pathogenic/likely pathogenic gene variant (PV/LPV) would have been missed if the testing had been based on 2019 NCCN guidelines (v3, 2019).^2–4^

In 2023, the NCCN criteria were expanded to include universal testing of all breast cancer patients with triple-negative disease patients and all patients age 50 years or younger at diagnosis.^5^ In 2024, the American Society of Clinical Oncology (ASCO) and the Society of Surgical Oncology (SSO) published joint recommendations for germline testing of all patients younger than 65 years with newly diagnosed breast cancer.^1^ Continued expansion of eligibility for germline testing will have an impact on the medical genetics capacity of every institution. In response, different approaches to using non-genetic health professionals for pretest counseling, also known as mainstream testing, have recently been studied or proposed.^6–9^

Kaiser Permanente Northern California (KPNC) currently treats more than 4000 breast cancer patients annually. A breast care coordinator (BCC) at each breast center does a standardized patient intake as part of the review at weekly case conferences of all early-stage patients. Eligible patients are referred to a genetic counselor (typically via a telehealth appointment) who reviews personal/family history and provides pre- and post-testing counseling to eligible patients. With the current workflow and staffing, expanding genetic testing eligibility to all breast cancer patients would not be scalable.

To reduce the impact of expanding genetic testing eligibility on genetics departments, four breast centers at KPNC piloted a new genetic testing pathway. This pathway was developed by a multidisciplinary team including medical genetics and precision medicine technology to offer testing at the time of intake by BCCs. Testing eligibility also was expanded to include all patients age 65 years or younger at the time of diagnosis (standard of care ≤ 45 years) and all triple-negative breast cancer patients (standard of care ≤ 60 years).^10–12^

This study aimed to compare testing uptake and results between breast cancer patients seen at our pilot sites with expanded eligibility and testing offered by BCCs and breast cancer patients seen at non-pilot sites and receiving standard of care.

## Methods

### Setting and Study Population

The study analyzed breast cancer patients who received a diagnosis at KPNC from 1 December 2021 to 31 June 2022. As a large integrated, community-based health care system, KPNC provides comprehensive primary and specialty care to approximately 4.4 million members in Northern California. The annual breast cancer volume at KPNC is approximately 4000 patients. The study excluded patients with stage IV cancer or with known PV/LPV in a hereditary cancer gene because these patients follow different care pathways.

### Data Collection

Patient demographics, tumor characteristics, and family history were abstracted from electronic medical records. Pathogenic and likely pathogenic variants (including low-penetrance variants and variants typically associated with recessive cancer susceptibility) were considered together as pathogenic variants. The family history information abstracted from the electronic medical records (EMRs) of patients at pilot sites age 46–65 years at diagnosis who had a PV/LPV in any gene was further reviewed manually to determine whether they would have met testing guidelines at non-pilot sites.

### Standard of Care for Germline Testing and Follow-up Evaluation During the Study Period

The KPNC health care system has 21 hospitals and 14 breast centers. Each center has a BCC who works with the patient and her breast treatment team for coordination of care.

#### Standardized Intake by the BCC

**Fig. 1:**
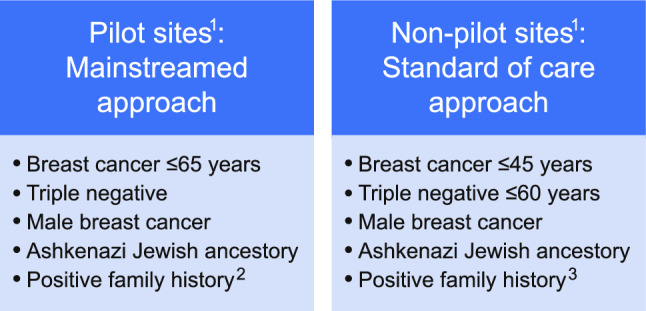
Testing criteria for pilot and non-pilot sites. 1. The pilot sites included 4 medical centers, and the non-pilot sites included 10 medical centers. 2. Defined as ≥ 3 first- or second-degree relatives with any of the following: female breast cancer diagnosed at age ≤ 50 years, or pancreatic, ovarian, or male breast cancer diagnosed at any age. 3. Defined as ≥ 1 first- or second- degree relatives with female breast cancer diagnosed at ≤ 50 years, or ≥ 2 first- or second-degree relatives with pancreatic, ovarian, or male breast cancer diagnosed at any age.

After patients are informed about their breast cancer diagnosis, they are contacted by the BCC. This initial intake encounter with the BCC includes standardized questions about family history designed with medical genetics to target features of hereditary breast and ovarian cancer syndrome as well as other heritable cancer predisposition syndromes (Fig. 1). The BCC also schedules an appointment for the patient with the patient’s treatment team. The BCC makes a referral to the genetics department for patients meeting age and family history criteria at this intake encounter, and this is done before the patient’s meeting with the surgeon or oncologist.

#### Tumor Board Review

The tumor board typically includes surgeons, oncologists, pathologists, radiologists, radiation oncologists, and genetic counselors. Each patient is reviewed at weekly breast tumor board meetings. Genetic counselors review each patient’s genetic testing eligibility as a part of the case conference. For a small number of patients, review of family history information at the tumor board results in a referral to genetics, and in these situations, a patient might meet with genetics after meeting with the surgeon or oncologist.

#### Germline Testing Guidelines

Referral of breast cancer patients for genetic testing is standardized throughout the KPNC system. During the study period, the guidelines for referral to receive pre-test counseling by a genetics provider included breast cancer patients with a diagnosis at age 45 years or younger, triple-negative disease diagnosed at or before age 60 years, male sex assigned at birth, Ashkenazi Jewish ancestry, or a family history that includes at least one first- or second-degree relative with female breast cancer diagnosed at or before age 50 years or two or more first- or second-degree relative with pancreatic, ovarian, or male breast cancer diagnosed at any age (Fig. 1).

#### Referral to Genetics and Communication of Results

Breast cancer patients meeting KPNC genetics referral guidelines are sent to genetics for pre-test counseling. Appointments are scheduled within 7 days after a new breast cancer diagnosis. Genetic counselors meet with referred patients in the patients’ preferred format (almost always by video) for individual pre-test counseling. Genetic counselors communicate the results to tested patients and provide interpretation, assessment, documentation, and a follow-up plan as necessary (with physician geneticist consultation as needed).

#### Germline Panel

The Invitae custom 62-gene panel (Invitae Corporation, San Francisco, CA, USA) for germline genetic testing was used in breast and other cancers. This panel was designed to provide comprehensive coverage across the most common cancer types and hereditary tumor predisposition syndromes with sufficient evidence and established care guidelines. The DNA analysis included next-generation sequencing with polymerase chain reaction (PCR) and multiplex ligation-dependent probe amplification (MLPA) as needed. The panel was stratified into high-risk breast cancer genes (*BRCA1, BRCA2, CDH1, PALB2, PTEN, TP53, STK11*), moderate-risk breast cancer genes (*ATM, BARD1, CHEK2, NF1, RAD51C, RAD51D*), and non-breast cancer genes (*APC,AXIN2,BAP1,BMPR1A,BRIP1, CDK4, CDKN1B, CDKN2A, CTNNA1, DICER1, EGLN1, EPCAM, FH, FLCN, GREM1,HOXB13, KIF1B, KIT, MAX,MEN1,MET,MITF,MLH1,MSH2, MSH3, MSH6, MUTYH, NF2, NTHl1, PDGFRA,PMS2,POLD1,POLE, POT1, RB1,RECQL4,RET, SDHA, SDHAF2, SDHB, SDHC, SDHD, SMAD4, SMARCA4,TMEM127, TSC1,TSC2,VHL,WT1*).

### Germline Testing, Workflows, and Follow-up Evaluation at Pilot Sites

Four breast cancer centers volunteered to be pilot sites. Each pilot site had a BCC who participated in the pilot study. Two BCCs were registered nurses, and two were nurse practitioners. The BCCs had been working at KPNC for an average of 4 years. These four coordinators participated in education on providing patients with pre-test informed consent discussions. They reported consistent positive feedback from patients, which provided further encouragement for this extra effort on behalf of the care team. All BCCs used the order set, and some incorporated the custom dashboard developed in the Epic KP HealthConnect electronic medical record system.

#### Standardized Intake by the BCC at Pilot Sites

The BCCs followed the same intake process described earlier for standard of care, but instead of referring patients meeting germline testing guidelines to genetics, the BCCs provided information on genetic testing and ordered it for eligible and consenting patients. These patients were asked to watch a brief educational video on genetic testing (internally produced). Patients who had complex family histories and/or wanted additional information could be referred to genetics for pre-test counseling.

#### Tumor Board Review at Pilot Sites

Review of cases by the tumor board at pilot sites was the same as described earlier for standard of care. Similarly, for a small number of patients, review of family history information at the tumor board resulted in a referral to genetics for pre-test counseling.

#### Germline Testing Guidelines at Pilot Sites

The guidelines at pilot sites differed from the standard of care described earlier, with testing recommended for breast cancer patients with a diagnosis at age 65 years or younger, triple-negative disease diagnosed at any age, male sex assigned at birth, Ashkenazi Jewish ancestry, or a positive family history, defined has having three or more first- or second-degree relatives with any of the following: female breast cancer diagnosed at or before age 50 years or pancreatic, ovarian, or male breast cancer diagnosed at any age (Fig. 1).

#### Ordering and Documentation of Genetic Tests by BCCs at Pilot Sites

At the time germline testing was offered, the BCCs documented patient consent using a new order set created within the EMR, and instructed patients to have their blood drawn. The BCCs also documented family history information collected using the same standardized questions as in standard of care.

#### Communication of Test Results at Pilot Sites

Results of genetic testing were tracked by the BCCs, with the option to use a custom dashboard that captures patients with active orders. If the test was negative and the patient did not report a significant family history of cancer (applicable to the majority), the BCC communicated the result and documented this in the chart using templates created in partnership with medical genetics. Patients were referred to genetic counseling if the test identified a PV/LPV or variant of uncertain significance (VUS), or the test was negative, but the patient had a significant family history. Genetic counselors communicated the results and provided interpretation, assessment, documentation, and any follow-up plan (with or without physician geneticist consultation as needed).

### Statistical Analysis

All data were summarized using means, medians, range, and standard deviations for continuous variables and frequencies and percentages for categorical variables. All data analyses were performed using SAS software version 9.4 (SAS Institute Inc, Cary NC, USA). The Institutional Review Board at KPNC determined that the protocol was exempt on 11 October 2022.

## Results

Patient and disease characteristics were generally similar at the pilot and non-pilot sites (Table 1). At both the pilot sites (502 patients seen during the study period) and the non-pilot sites (1792 patients seen during the study period) sites, the median age of breast cancer diagnosis was 64 years, and approximately half of the population was non-Hispanic white.

**Table 1:** Patient and disease characteristics at pilot and non-pilot sites

Characteristic	Pilot	Non-pilot
	*n* (%)^a^	*n* (%)^a^
Overall	502	1792
Age at diagnosis (years)		
Median	64	64
≤ 45	46 (9.2)	178 (9.9)
46–65	214 (42.6)	767 (42.8)
≥ 66	242 (48.2)	847 (47.3)
Family hx of breast cancer^b^		
Yes	221 (44.0)	670 (37.4)
No	281 (56.0)	1122 (62.6)
Family hx of ovarian cancer^b^		
Yes	41 (8.2)	100 (5.6)
No	461 (91.8)	1692 (94.4)
Family hx of colon cancer^b^		
Yes	89 (17.7)	283 (15.8)
Race/ethnicity		
Non-hispanic white	264 (52.6)	959 (53.5)
Hispanic	67 (13.3)	259 (14.5)
Asian	96 (19.1)	441 (24.6)
Black	67 (13.3)	115 (6.4)
Unknown	8 (1.6)	17 (0.9)
Tumor subtype		
Triple-negative	43 (8.6)	123 (6.9)
Tumor stage		
DCIS	72 (14.3)	273 (15.2)
Invasive cancer	421 (83.9)	1478 (82.5)
Unknown	9 (1.8)	41 (2.3)

The study population includes members of Kaiser Permanente Northern California diagnosed with non-metastatic breast cancer from December 2020 to June 2021. The source of data was the electronic medical record.

*hx* history, *DCIS* ductal carcinoma in situ

^a^Percentages are column percentages

^b^Includes 1st- and 2nd-degree relatives

During the study period, the diagnosis was at a pilot site for 1 male breast cancer patient and at a non-pilot site for 13 patients. A family history of breast cancer was slightly more common among the patients at the pilot sites (44% vs 37%). The pilot sites had a slightly lower percentage of patients with ductal carcinoma in situ (DCIS) (14.3% vs 15.2%) and a slightly higher percentage of patients with triple-negative disease (8.6% vs 6.9%).

The percentage of all the patients undergoing germline genetic testing was approximately two times higher at the pilot sites than at the non-pilot sites (61.6% vs 31.7%; Table 2), and the percentage tested was higher across all age groups and among patients with triple-negative breast cancer. The percentage tested decreased with increasing age at diagnosis at both the pilot and non-pilot sites. Although genetic testing was offered to all patients age 65 years or younger at pilot sites, the testing rate was somewhat higher among those age 45 years or younger than among those 46–65 years of age (97.5% vs. 83.2%). In addition to the substantially increased percentage of patients tested at the pilot sites, the median time from biopsy to test result was substantially shorter at the pilot sites (22 vs 33 days).

**Table 2:** Patients undergoing genetic testing by age group and time from diagnosis to testing

	Pilot sites			Non-pilot sites		
	Tested	Not tested	Total	Tested	Not tested	Total
	*n* (%)^a^	*n* (%)^a^	*n*	*n* (%)^a^	*n* (%)^a^	*n*
Overall	309 (61.6)	193 (38.4)	502	568 (31.7)	1224 (68.3)	1792
Age at diagnosis (years)						
< 45	44 (95.7)	2 (4.3)	46	154 (86.5)	24 (13.5)	178
46–65	178 (83.2)	36 (16.8)	214	262 (34.2)	505 (65.8)	767
> 66	87 (36.0)	155 (64.0)	242	152 (17.9)	695 (82.1)	847
Tumor subtype						
Triple-negative	37 (86.0)	6 (14.0)	43	67 (54.5)	56 (45.5)	123
Time from Dx to test result						
Median (days)	22	NA		33	NA	
Mean (days)	29	NA		39	NA	

The study population included members of Kaiser Permanente Northern California with a diagnosis of non-metastatic breast cancer from December 2020 to June 2021. The source of the data was the electronic medical record.

*Dx* diagnosis

^a^Percentages are row percentages

Among tested patients at the pilot and non-pilot sites, 8.7% versus 9.7% had a PV/LPV identified in one or more genes included in the full 61-gene panel, and 5.8% versus 5.1% had a PV/LPV identified in a high- or moderate-risk breast cancer-related gene (Table 3). Among all the patients (tested and untested) at the pilot and non-pilot sites, 5.4% versus 3.1% had a PV/LPV identified in one or more genes in the full panel, and 3.4% versus 1.6% had a PV/LPV identified in a breast cancer-related gene (Table 4). At both the pilot and non-pilot sites, the patients age 45 years or younger at diagnosis and the patients with triple-negative tumors had the highest prevalence of PV/LPVs for any gene or for high/moderate-risk breast cancer-related genes.

**Table 3:** PV/LPVs among tested patients

	Pilot sites		Non-pilot sites	
	Any gene % (*n*)^c^	HR^a^/MR^b^ gene % (*n*)^c^	Any gene% (*n*) % (*n*)^c^	HR^a^/MR^b^ gene % (*n*)^c^
Overall	8.7 (27/309)	5.8 (18/309)	9.7 (55/568)	5.1 (29/568)
Age at diagnosis				
≤ 45 years	15.9 (7/44)	13.6 (6/44)	12.3 (19/154)	7.1 (11/154)
46–65 years	7.9 (14/178)	5.6 (10/178)	8.8 (23/262)	5 (13/262)
≥ 66 years	6.9 (6/87)	2.3 (2/87)	8.6 (13/152)	3.3 (5/152)
Triple-negative	16.2 (6/37)	8.1 (3/37)	17.9(12/67)	8.96 (6/67)

The study population included members of Kaiser Permanente Northern California with a diagnosis of non-metastatic breast cancer from December 2020 to June 2021. The source of the data was the electronic medical record

*PV* pathogenic variant, *LPV* likely pathogenic variant, *HR* high risk for breast cancer, *MR* moderate risk for breast cancer

^a^HR: *BCRA1, BRCA2, CDH1, PALB2, PTEN, TP53, STK11*

^b^MR: *ATM, BARD1, CHEK 2, NF1, RAD51C, RAD51D*

^c^The nos. in the parenthesis are the numerator and denominator for the corresponding percentage

**Table 4:** PV/LPVs among all patients

	Pilot site		Non-pilot site	
	Any gene % (*n*)^c^	HR^a^/MR^b^ gene % (*n*)^c^	Any gene % (*n*)^c^	HR^a^/MR^b^ gene % (*n*)^c^
Overall	5.4 % (27/502)	3.6 (18/502)	3.1 (55/1792)	1.6 (32/1792)
Age at diagnosis				
≤ 45 years	15.2 (7/46)	13 (6/46)	10.7 (19/178)	6.2 (11/178)
46–65 years	6.5 (14/214)	4.7 (10/214)	3 (23/767)	1.7 (13/767)
≥ 66 years	2.5 (6/242)	0.8 (2/242)	1.5 (13/847)	0.6 (5/847)
Triple-negative	13.95 (6/43)	6.97 (3/43)	9.76 (12/123)	4.9 (6/123)

The study population included members of Kaiser Permanente Northern California with a diagnosis of non-metastatic breast cancer from December 2020 to June 2021. The source of the data was the electronic medical record

*PV* pathogenic variant, *LPV* likely pathogenic variant, *HR* high risk for breast cancer, *MR* moderate risk for breast cancer

^a^HR: *BCRA1, BRCA2, CDH1, PALB2, PTEN, TP53, STK11*

^b^MR: *ATM, BARD1, CHEK 2, NF1, RAD51C, RAD51D*

^c^The nos. in the parenthesis are the numerator and denominator for the corresponding percentage

After review of the medical records for the patients with PV/LPVs identified at the pilot sites, 13 of 14 patients between ages 46 and 65 years would have been offered germline testing based on our current standard guidelines (i.e., NCCN 2024).

The percentage of patients identified with a VUS (Tables 5 and 6) was substantially higher than the percentage identified with a PV/LPV. Among the tested patients, 46.3% at the pilot sites and 54.2% at the non-pilot sites had a VUS. The percentage of patients with a VUS in any of the tested genes was highest among the patients with disease diagnosed at or before age 45 years. The prevalence of VUS in any tested gene was substantially higher than among breast cancer-related genes alone.

**Table 5:** VUS among tested patients

	Pilot site		Non-pilot site	
	Any gene % (*n*)^c^	HR^a^/MR^b^ gene % (*n*)^c^	Any gene % (*n*)^c^	HR^a^/MR^b^ gene % (*n*)^c^
Overall	46.3 (142/309)	14.2 (44/309)	54.2 (308/568)	17.95 (102/568)
Age at diagnosis				
≤ 45 years	63.6 (28/44)	25.0 (11/44)	61.7 (95/154)	17.5(27/154)
46–65 years	46.1 (82/178)	11.2 (20/178)	52.3 (137/262)	17.2 (45/262)
≥ 66 years	37.9 (33/87)	14.9 (13/87)	50 (76/152)	19.7 (30/152)

The study population included members of Kaiser Permanente Northern California with a diagnosis of non-metastatic breast cancer from December 2020 to June 2021. The source of the data was the electronic medical record

*VUS* variant of uncertain significance, *HR* high risk for breast cancer, *MR* moderate risk for breast cancer

^a^HR: *BCRA1, BRCA2, CDH1, PALB2, PTEN, TP53, STK11*

^b^MR: *ATM, BARD1, CHEK 2, NF1, RAD51C, RAD51D*

^c^The nos. in the parenthesis are the numerator and denominator for the corresponding percentage

**Table 6:** VUS among all patients

	Pilot site		Non-pilot site	
	Any gene % (*n*)^c^	HR^a^/MR^b^ gene % (*n*)^c^	Any gene % (*n*)^c^	HR^a^/MR^b^ gene % (*n*)^c^
Overall	28.3 (142/502)	8.8 (44/502)	17.2 (308/1792)	5.7 (102/1792)
Age at diagnosis				
≤ 45 years	60.9 (28/46)	23.9 (11/46)	53.4 (95/178)	15.2 (27/178)
46–65 years	38.3 (82/214)	9.3 (20/214)	17.9 (137/767)	5.9 (45/767)
≥ 66 years	13.6 (33/242)	5.4 (13/242)	9.0 (76/847)	3.5 (30/847)

The study population included members of Kaiser Permanente Northern California with a diagnosis of non-metastatic breast cancer from December 2020 to June 2021. The source of the data was the electronic medical record

VUS, variant of uncertain significance; HR, high risk for breast cancer; MR, moderate risk for breast cancer

^a^HR: *BCRA1, BRCA2, CDH1, PALB2, PTEN, TP53, STK11*

^b^MR: *ATM, BARD1, CHEK 2, NF1, RAD51C, RAD51D*

^c^The nos. in the parenthesis are the numerator and denominator for the corresponding percentage

The specific genes in which a PV/LPV was identified are presented in Table 7. At the pilot sites, PV/LPVs in breast cancer genes were most commonly identified in *ATM, BRCA2*, and *BRCA1,* whereas at the non-pilot sites, they were most commonly identified in *BRCA2, BRCA1, and PALB2*. For non-breast cancer-related genes, PV/LPVs were most commonly identified in *MUTYH* (mono-allelic) at both the pilot and non-pilot sites.

**Table 7:** Patients identified with PV/LPVs in specific genes

Gene risk group	Gene	Pilot *n* (%)	Non-pilot *n* (%)	Total
High-risk breast cancer genes	*BRCA1*	3 (1.0)	8 (1.4)	11
	*BRCA2*	4 (1.3)	9 (1.6)	13
	*PALB2*	2 (0.6)	5 (0.9)	7
	*TP53*	1 (0.3)	1 (0.2)	2
	TOTAL	10 (3.2)	23 (4.0)	
Moderate-risk breast cancer genes	*ATM*	6 (1.9)	3 (0.5)	9
	*BARD1*		2 (0.4)	2
	*CHEK2*	2 (0.6)		2
	*RAD51D*		1 (0.2)	1
	TOTAL	8 (2.6)	3 (1.1)	
Non-breast cancer genes	*APC*	1 (0.3)	1 (0.2)	2
	*BRIP1*		2 (0.4)	2
	*CDKN2A*		3 (0.5)	3
	*FH*	1 (0.3)	3 (0.5)	4
	*MITF*		1 (0.2)	1
	*MSH3*		1 (0.2)	1
	*MUTYH*	5 (1.6)	8 (1.4)	13
	*NTHL1*		1 (0.2)	1
	*PMS2*	1 (0.3)	2 (0.4)	3
	*POLE*		1 (0.2)	1
	*RECQL4*	1 (0.3)		1
	*SDHA*		1 (0.2)	1
	*SDHC*		1 (0.2)	1
	*VHL*		1 (0.2)	1
	TOTAL	9 (2.9)	26 (4.6)	

*PV* pathogenic variant, *LPV* likely pathogenic variant

Although we did not have information on the patients seen by genetics but not tested, we can estimate the number of patient visits with genetics based on testing pathways and workflows for the pilot and non-pilot sites. Thus, we can assume that at the non-pilot sites, all the patients tested had contact with genetics both before and after testing. At the pilot sites, we can assume that the patients tested and found to have a PV/LPV or VUS had contact with genetics after but not before testing. Under these assumptions, 33.7% of all the patients at the pilot sites (29 with PV/LPV plus 142 with VUS; *n* = 502) and 31.7% of all the patients at the non-pilot sites (568 tested; *n* = 1792) had contact with genetics. At the non-pilot sites, we also assumed that those tested had two contacts with genetics (before and after testing). Thus, even if we assumed that up to 20% of the patients at the pilot sites (*n* = 100) were seen at genetics for pre-test counseling (e.g., because of a complex family history or referral by a tumor board), the number of visits per total patients at the pilot sites still would have been lower (0.54 visits per total patients) than at the non-pilot sites (0.58 visits per total patients) with testing of nearly twice the proportion of patients (61.6% vs 31.7%).

## Discussion

In this study of patients with early breast cancer diagnosed and treated in a community setting, we found that enabling BCCs to deliver mainstream germline genetic testing, with expansion of testing eligibility, resulted in almost a doubling of the percentage of patients undergoing germline testing (32–62% of all the patients) without increasing patient visits to genetic counselors. Although a higher percentage of total patients was identified with PV/LPVs in breast cancer-related genes (increase from 1.6% at the non-pilot sites to 3.6% at the pilot sites), a high percentage of the patients tested also were identified with a VUS (46.3% at the pilot sites and 54.2% at the non-pilot sites).

Since first described for ovarian cancer patients by George et al.^7^ in 2016, mainstream genetic testing uses nongenetic practitioners to provide pretest genetic counseling and consent. Many different models of mainstream germline genetic testing in cancer care have been proposed recently to meet increasing demand.^6,13–15^ Each model within mainstreaming genetic testing, such as models that simplify eligibility criteria or collaborate with genetics departments, shows how a different approach can influence infrastructure and stress different components necessary in building a program.

Recently, Bokkers et al.^13^ reported that mainstream testing using surgical oncologists and nurses at breast cancer centers in the Netherlands to order the germline testing is feasible. Our pilot study used nurses and nurse practitioners who were breast care coordinators (BCCs) to provide pretest counseling. Our BCCs are embedded in each breast program, performing intake interviews with breast cancer patients who are reviewed later at weekly breast case conferences. Our findings suggest that integrating genetic testing into our initial intake, even in a health system with no charge to the patient for genetic testing, may remove one more barrier by decreasing the number of appointments for testing.^16,17^ Confirming other mainstream testing study results, we also were able to decrease the median time between biopsy and test result (22 vs 33 days).^6,15^ In addition, mainstream testing appeared able to substantially increase the percentage of patients tested without increasing the percentage of patients seen by genetics,

Many studies have shown that a high percentage of breast cancer patients is identified as having a VUS when undergoing multigene panel testing. Kurian et al.^18^ examined germline testing in California and Georgia among breast and ovarian cancer patients from 2012 to 2019 and found that the percentage with VUS doubled over time (11.2% in 2013 to 26.8% in 2017). The study by Beitsch et al.^2^ in a community setting showed a VUS rate of 54.2%, and although the participating physicians counseled their patients about VUSs, they did not use VUS to change patient management. Our study, in which patients were tested with a 62-gene panel, also showed a high percentage with VUS (46.3% vs 54.2% among tested patients at the pilot vs the non pilot sites).

Since the start of our pilot study, the MAGENTA clinical trial showed that omitting individualized pretest counseling for all participants and post-test counseling for those without PV during remote genetic testing was not inferior regarding post-test distress, providing an alternative care model for genetic risk assessment.^17^ After evaluating our data on VUS and the MAGENTA trial data, we developed a communication pathway that enables non-genetic counselors to communicate the VUS and non-actionable PV results to our patients.

To our knowledge, our study is the first in a community hospital setting to offer a hybrid model that includes testing all breast cancer patients age 60 or younger or younger than 65 years, as advocated by Yadav et al.^12^ and Desai et al.^11^ Universal testing up to age 65 years or younger has been shown to increase the sensitivity for pathogenic variants in *BRCA1* or *BRCA2* to more than 98%.^12^ The expanded age criteria detected more than twice as many PV/LPVs in breast cancer-related genes (3.6% vs 1.6%) and 1.7 times as many PV/LPVs in any of the tested genes (5.4% vs.3.1%). We found that 13 of 14 patients ages 46–65 years at the pilot sites identified with PV/LPVs would have been offered germline testing based on our standard guideline. However, this did likely reduce the amount of time from breast cancer diagnosis to test result but did reduce the number of medical appointments the patient needed to have before getting genetic testing.

One strength of our study was the diverse population, which closely reflected the sociodemographic of California and was approximately 50% non-Hispanic white. In addition, because KPNC members get virtually all their care within the health plan, we had very complete information on germline testing and results.

A limitation of this study was that the testing pathway might not be generalizable to non-integrated health care settings. Compared with 35% of the NCCN-eligible patients referred to genetics in some community settings,^19^ findings from our study and others^20,21^ indicate that the current standard of care at KPNC already refers a high proportion of breast cancer patients (e.g., 86.5% of patients ≤ 45 years were tested at the non-pilot sites).

## Conclusion

Simplification of eligibility by offering mainstream germline hereditary cancer genetic testing to all patients age 65 years or younger through BCCs detected a higher prevalence of PV/LPVs than the standard guideline-based testing while preserving the limited resources of our genetic counseling departments. Reducing the impact of expanded testing on genetic counseling further will require consideration of more efficient ways to communicate VUS and non-actionable PV/LPVs.
